# Early Rise of Serum hCG in Gestational Diabetes Mellitus Women With Live Birth Through *In Vitro* Fertilization Procedure

**DOI:** 10.3389/fendo.2022.724198

**Published:** 2022-02-15

**Authors:** Wei Wu, Li-Feng Zhang, Yi-Ting Li, Tian-Xiao Hu, Dan-Qing Chen, Yong-Hong Tian

**Affiliations:** ^1^ Department of Reproductive Endocrinology, Women’s Hospital, Zhejiang University School of Medicine, Hangzhou, China; ^2^ Department of Endocrinology, Chinese PLA 903rd Hospital (Former Chinese PLA 117th Hospital), Hangzhou, China; ^3^ College of Life Science, Zhejiang Chinese Medical University, Hangzhou, China; ^4^ Department of Obstetrics, Women’s Hospital, Zhejiang University School of Medicine, Hangzhou, China; ^5^ Key Laboratory of Reproductive Genetics, Ministry of Education, Hangzhou, China

**Keywords:** hCG, gestational diabetes mellitus (GDM), *in vitro* fertilization, live birth, reproduction

## Abstract

Gestational diabetes mellitus (GDM) is one of the most common complications of pregnancy. The characteristics of early human chorionic gonadotropin (hCG) levels and the rise pattern in patients with GDM after *in vitro* fertilization (IVF) are unclear. The present investigation was a retrospective cohort analysis of eligible viable pregnancies achieved through IVF in the authors’ hospital between October 2015 and June 2020. The characteristics of initial hCG concentration and the rise pattern in patients with GDM after IVF, and the difference between those of normoglycemic pregnant women, were explored. Using random-effects models, the preferred pattern to describe the increase in log hCG was a quadratic. When gestational age was within 39 days, the linear model adequately characterized the profile, and the average slope was 0.173, yielding a predicted increase of 1.55 (55%) in 1 day and 3.11 (211%) in 2 days. Absolute hCG values—but not the rate of rise—were significantly higher in double embryo transfers and twin pregnancies. Curves reflecting hCG rise from the GDM and non-GDM groups did not differ substantially. The proportion of patients with low initial hCG values (16 days post-oocyte retrieval <100 mIU/ml) was higher in the GDM group (5% vs. 2.09%), although the difference was not statistically significant. Early hCG rise in pregnant women after IVF—whether GDM or non-GDM—could be characterized by quadratic and linear models. However, hCG values on days 14 and 16 post-oocyte retrieval in the GDM group were lower than those in the non-GDM group, with the exception of twin pregnancies. Low hCG values in early pregnancy may be a clue to help predict GDM in the subsequent gestation period.

## Introduction

Human chorionic gonadotropin (hCG) is a glycoprotein secreted by human blastocysts and can be detected in the plasma of pregnant women approximately 6–8 days after fertilization (1). When ultrasonography is non-diagnostic, an increase in hCG level in early pregnancy is very important for clinicians to judge whether the pregnancy is viable. Currently, clinicians largely rely on a normal “doubling time” (66% rise in 2 days) (2). However, a study involving a large cohort of women with spontaneously conceived pregnancies and symptoms of pain or bleeding reported that the minimal increase in serial hCG values in those with a viable intrauterine pregnancy was “slower” than previously reported, with the slowest increase being 24% at 1 day and 53% at 2 days (3).

The early rise pattern of hCG level cannot only be used to estimate the likelihood of viable pregnancy but can also indicate the possibility of pregnancy complications in some cases. Some studies have demonstrated a significant association between low hCG levels and adverse pregnancy outcomes, including fetal loss (4), intrauterine growth restriction, and low birth weight (4, 5), while others, in contrast, have not reported such associations (6, 7). These conclusions are based on natural pregnancy and cannot be directly extended to pregnancies achieved through assisted reproductive technology. As such, Chung et al. (8) conducted a comprehensive study investigating the hCG profile of viable pregnancies conceived through *in vitro* fertilization (IVF). The results indicated that it was comparable to previous estimates of symptomatic spontaneous conceptions (8). However, this study only examined the rate of hCG rise associated with all viable pregnancies achieved through IVF without considering subsequent maternal complications including gestational hypertension or gestational diabetes mellitus (GDM). It is well known that some pregnancy complications are associated with abnormal early hCG values in spontaneous pregnancies (4, 5). Few similar studies have examined pregnancy achieved through IVF procedures; nevertheless, significant differences in serum hCG levels among women with and without obesity after single embryo transfer (ET) have been reported in a recent study (9).

GDM is one of the most common medical complications of pregnancy and represents a major risk factor for both adverse maternal–fetal (10, 11) and long-term maternal (12, 13) complications. A previous study suggested that high free hCG levels in the first trimester (11–13^+6^ weeks) of pregnancy decreased the risk for GDM (14). However, to the best of our knowledge, whether absolute hCG levels or the rate of rise in hCG levels are associated with later development of GDM in women who undergo IVF is not known. Therefore, it is necessary to determine whether the clinical rules derived from all populations are applicable to the unique patient population that achieves viable pregnancies through IVF technology.

The purpose of our study was to describe increases in serum hCG levels in a large cohort of pregnant women who conceived through IVF and later developed GDM and to evaluate potentially influential factors to predict pregnancy complications.

## Materials and Methods

This study was approved by the Women’s Hospital of the Zhejiang University School of Medicine (Hangzhou, Zhejiang Province, China). Data were collected from the medical records of women who underwent fresh or frozen ET and achieved live births between October 2015 and June 2020 in the authors’ reproductive center. Autologous IVF or intracytoplasmic sperm injection cycles with transfer of 1 or 2 embryos on day 2, 3, or 5 were included.

All pregnancies achieved through IVF, performed between October 2015 and June 2020, were considered for inclusion in this study. Pregnancies with live births of 1 to 2 infants were eligible. Values that were measured at intervals of ≥24 h and ≤7 days were included in the analysis. Serum hCG measurements obtained before 39 days’ gestation (25 days post-oocyte retrieval) had the optimal clinical value because during this period, ultrasound was non-diagnostic and, as a result, monitoring of serial hCG levels was clinically most appropriate (8). Thus, the analysis was limited to hCG measurements obtained before 25 days post-oocyte retrieval. Except for GDM, those with other conditions, such as maternal diabetes and hypertension before pregnancy, gestational hypertension, thyroid disease, and heart disease, were not included in the study. Additional inclusion criteria required that patients had ≥1 serum hCG level(s) documented in the hospital information system of the authors’ agency. In total, 1,005 cases fulfilled the inclusion criteria and contributed a total of 1,839 hCG values.

At 24–28 weeks’ gestation, screening for GDM was performed using a 75-g oral glucose tolerance test (OGTT). Diagnosis of GDM was made in accordance with the International Association of Diabetes and Pregnancy Study Groups glycemic cutoff values [fasting value ≥92 mg/dl (5.1 mmol/L), 1-h post-glucose load ≥180 mg/dl (10 mmol/L), and 2-h post-glucose load ≥153 mg/dl (8.5 mmol/L)] (15). Serum hCG concentrations were determined using a commercially available electrochemiluminescence immunoassay (Cobas e601, Roche Diagnostics, Mannheim, Germany). Results are expressed as mIU/ml using the third International Reference Preparation. Patient demographic information and details of maternal age, body mass index (BMI), fasting blood glucose level, OGTT 1-h/2-h post-glucose load, birth weight of newborn, first test of embryonic age, number of observations, and number of ETs were extracted from patient medical records. The curves created by hCG determination, limited to 39 gestational ages, were evaluated. For all analyses, time was measured according to embryonic age (days post-oocyte retrieval), and all hCG values were transformed to the natural log scale to better approximate a normal distribution. Using embryonic age as the independent variable and log hCG as the dependent variable, quadratic and linear models were explored. Different curves were used to analyze the difference in hCG increase between the GDM and non-GDM groups. Multivariable analysis was used to analyze factors that may affect initial hCG values, including maternal age, embryonic age, BMI, number of gestational sacs, number of embryos transferred, and fresh or frozen ET. From the final model, population average values for increase or slope, standard errors, and upper and lower confidence bounds for the rate of increase in log hCG were estimated, which assumed that log hCG followed a normal distribution. In addition to the average values of increase, corresponding upper and lower confidence estimates (percentiles) for the rate of increase are also presented. Primary data management and analyses were performed using SPSS version 20 (IBM Corporation, Armonk, NY, USA).

A subanalysis limited to patients with serum hCG values checked at least twice was performed (including 740 patients with 1,574 hCG values). For patients in whom hCG levels were not checked exactly on day 14 or day 16 post-oocyte retrieval, the values were calculated using the log-linear rate of hCG increase in early pregnancy according to a previous study (3, 9). Briefly, the difference between the log of the first two hCG values was calculated and divided by the number of days between them. The result was multiplied by the number of days from day 14 or day 16, at which point the first hCG value was collected. This value was then added (if the first hCG level had been checked after day 14 or 16) or subtracted (if the first hCG had been checked before day 14 or 16) to the log of the first available value, which was then exponentiated. The proportion of patients with low initial hCG values (defined as hCG on day 16 post-oocyte retrieval <100 mIU/ml) was also calculated. The rates of low rise (mean 1 day hCG rise <55%), which were derived from the data (**Table 2**), in the GDM and non-GDM groups were also analyzed. Additionally, multivariable analysis was used to analyze factors that affected fasting glucose value and 1-h and 2-h post-glucose load in the OGTT in all GDM and non-GDM cases.

## Results

The final study sample of 1,005 pregnant women contributed a total of 1,839 hCG values: 305 patients with 604 hCG values in the GDM group and 700 patients with 1,235 hCG values in the non-GDM group. Descriptive data and the characteristics of the two groups are summarized in **Table 1**.

**Table 1:** Basic characteristics of the GDM and non-GDM groups.

Characteristic	Group		*p* value
	GDM (n = 305)	Non-GDM (n = 700)	
Age (years)	32.28 ± 3.62	31.79 ± 3.46	0.063
Body mass index (kg/m^2^)	21.79 ± 2.42	21.42 ± 2.51	0.037
Fasting blood glucose (mmol/L)	4.74 ± 0.52	4.40 ± 0.31	<0.001
1-h blood glucose (mmol/L)	10.24 ± 1.46	7.72 ± 1.29	<0.001
2-h blood glucose (mmol/L)	8.96 ± 1.43	6.55 ± 1.02	<0.001
Birth weight of newborn (g)	3,033.51 ± 594.60	3,071.66 ± 614.45	0.564
First test of embryonic age (days)	15.38 ± 1.78	15.4 ± 1.85	0.678
Average number of observations	1.98 ± 0.73	1.76 ± 0.48	<0.001
Average embryos transferred	1.74 ± 0.44	1.82 ± 0.38	0.006
Proportion of patients with low initial hCG*, %	5.00	2.09	>0.05
Rate of low rise^◊^, %	52.1	48.9	0.225

Data presented as mean ± SD or % unless otherwise indicated.

*Proportion of patients with human chorionic gonadotropin (hCG) on day 16 post-oocyte retrieval <100 mIU/ml.

**◊**hCG 1 day rise <55% based on the data (**Table 2**).

GDM, gestational diabetes mellitus.

**Figure 1:**
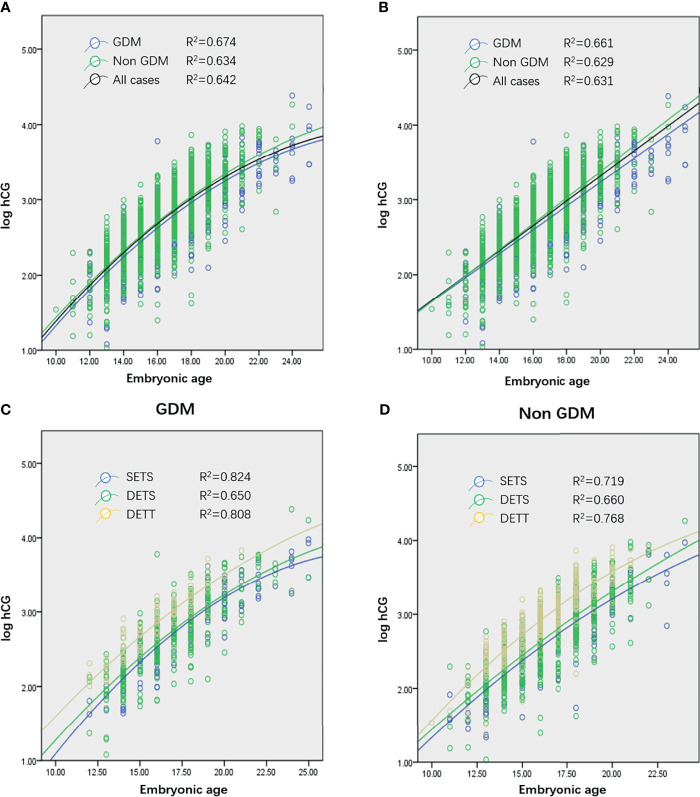
**(A, B)** Quadratic curves **(A)** and linear plot **(B)** generated from serial log human chorionic gonadotropin (hCG) concentrations of women who conceived through *in vitro* fertilization (IVF) and ultimately achieved live births from all cases (1,005 patients, 1,839 observations), gestational diabetes mellitus (GDM) cases (305 patients, 604 observations), and non-GDM cases (700 patients, 1,235 observations). **(C, D)** Curves generated from single embryo transfer and singleton pregnancy (SETS; 196 patients, 394 observations), two embryo transfers and singleton pregnancy (TETS; 542 patients, 980 observations), and two embryo transfers and twin pregnancy (TETT; 261 patients, 447 observations) cases in GDM **(C)** and non-GDM **(D)** groups. No curve of single embryo transfer and twin pregnancy was generated because only 9 patients were recruited in the present study.

The transformed log hCG was tested using a single-sample Kolmogorov–Smirnov test and proven to be normally distributed (z = 0.878, *p* = 0.423). Using a scatter plot to establish a fitting curve, the optimal pattern (with a maximum R^2^) to describe the rise of log hCG from all GDM and non-GDM cases was quadratic (**Figure 1A**, black curve). To explore the difference in the initial hCG level and the rate of increase between women with and without GDM, all cases were divided into two subgroups: GDM and non-GDM. Statistical analysis revealed no difference in the increase in hCG between the two groups (**Figures 1A, B** **)**.

Multivariable analysis revealed that absolute values of log hCG were significantly influenced by the number of gestational sacs (*p* < 0.001) and the number of ETs (*p* < 0.001). Further study was conducted to explore whether these two factors had the same effect on hCG increase in the population. The results revealed that, in both the GDM and non-GDM groups, the number of gestational sacs and the number of embryos transferred (**Figures 1C, D** **)** did not affect the rate of rise of hCG levels.

Overall, the preferred model included a fixed quadratic effect and a random linear effect for embyonic age, as well as fixed effects for the number of gestational sacs and number of embryos transferred. The population average profile for log hCG per day of embryonic age (days) is described by the following equation:


$$\begin{array}{c} \log\text{ HCG}=0.347\;(\text{days})-0.005\;({\text{day}}^{2})+0.282\;(\text{gestational sac})\\ +0.073\;(\text{number of embryos transferred})-2.048 \end{array}$$

Several studies revealed that when the analysis was limited to measurements obtained before 39 days’ gestation, the log hCG profile can be adequately characterized by a linear model (3, 8). In the present study, it was also found that the linear model adequately described the profile of log hCG (**Figure 1B**). Using the linear model, the equation is as follows:


log hCG=0.173 (days)+0.284 (gestational sac)+0.083 (number of embryos transferred)−0.622

In this model, log hCG levels were significantly higher among twins compared to singletons (2.90 ± 0.44 vs. 2.70 ± 0.49; *p* < 0.001) and higher among women with two embryos transferred (2.77 ± 0.48 vs. 2.67 ± 0.51; *p* = 0.033). Using this model, the expected rates of increase in hCG values were derived, as shown in **Table 2**. On average, patients experienced a relative increase of 1.55 [99% confidence interval (CI) 1.55–1.56] in 1 day and 3.11 (99% CI 3.10–3.11) in 2 days. Of the actual values observed, the slowest confirmed rise was 1.19 (19%) in 1 day and 2.38 (138%) in 2 days.

**Table 2:** Predicted relative increase in hCG levels over time.

	Slope of hCG rise	1 day later	2 days later	7 days later
1st percentile	0.112	1.19	2.38	8.34
5th percentile	0.126	1.29	2.58	9.04
Mean (99% CI)	0.173 (0.166–0.180)	1.55 (1.55–1.56)	3.11 (3.10–3.11)	10.87 (10.85–10.89)
95th percentile	0.231	2.27	4.54	15.87

CI, confidence interval; hCG, human chorionic gonadotropin.

After excluding 265 pregnant women who underwent hCG testing only once, 1,574 hCG values from 740 subjects remained. For patients in whom hCG levels were not checked exactly on day 14 or day 16 post-oocyte retrieval, the equivalent values were calculated using the log-linear rate of hCG increase in early pregnancy (3, 9). The results revealed that the initial hCG values in the non-GDM group were significantly higher than those in the GDM group at 14 days or 16 days post-oocyte retrieval, with the exception of twin pregnancies (**Table 3**). To further verify the reliability of the results, the original hCG values on day 14 (*n* = 204) and day 16 (*n* = 291) post-oocyte retrieval were selected from the population and compared with the calculated values. The results revealed that there was no significant difference in the mean values between original and calculated hCG on day 14 (231.67 ± 154.73 vs. 224.06 ± 149.66, *p* = 0.5) and day 16 (559.65 ± 374.32 vs. 553.22 ± 427.58, *p* = 0.813) post-oocyte retrieval.

**Table 3:** Comparison of hCG values between the GDM and non GDM groups, with hCG calculated on day 14 or day 16 post-oocyte retrieval.

	Group		*p* value
	GDM (n = 520)	Non-GDM (n = 1054)	
hCG (day 14 post-oocyte retrieval)			
Singleton	162.00 ± 103.96	196.87 ± 122.27	<0.001
Twins	348.87 ± 155.57	360.94 ± 173.43	0.554
Single transfer	152.07 ± 88.04	192.11 ± 133.12	0.001
Two embryo transfers	215.22 ± 148.00	251.99 ± 158.15	<0.001
**hCG (day 16 post-oocyte retrieval)**			
Singleton	388.76 ± 233.58	491.48 ± 416.50	<0.001
Twins	824.34 ± 330.21	898.71 ± 438.05	0.136
Single transfer	361.72 ± 208.28	502.13 ± 647.12	0.007
Two embryo transfers	514.64 ± 327.17	622.57 ± 393.13	<0.001

Data presented as mean ± SD unless otherwise indicated.

hCG, human chorionic gonadotropin; GDM, gestational diabetes mellitus.

Although the proportion of patients with low initial hCG values was higher in the GDM group, the difference was not statistically significant (5.00% vs. 2.09%, respectively; *p* > 0.05). The linear model of our study demonstrated a predicted mean increase of 1.55 (55%) in 1 day, and then we analyzed the rate of low rise (1 day hCG rise <55%) in the GDM and non-GDM groups. The results revealed no significant difference between the two groups (52.1% vs. 48.9%; *p* = 0.225).

Furthermore, multivariate analysis revealed that advanced maternal age and higher BMI were associated with elevated fasting values, 1-h post-glucose load, and 2-h post-glucose load in the OGTT. The initial hCG values on day 16 post-oocyte retrieval were weakly and negatively correlated with 1-h post-glucose load (*r* = -0.129, *p* < 0.001) and 2-h post-glucose load (*r* = -0.119, *p* < 0.001). Meanwhile, there was a very weak inverse correlation (*r* = -0.095, *p* < 0.001) between fasting glucose levels and hCG values on day 14 post-oocyte retrieval.

## Discussion

The primary objective of this study was to investigate the initial hCG level and the rate of increase in pregnant women who later developed GDM who delivered through IVF procedures and to evaluate whether the initial hCG levels and the rise pattern in pregnant women who later developed GDM differ from those without GDM. To the best of our knowledge, the present study is the first to focus specifically on the relationship between GDM and early hCG rise.

It has been suggested that the curve describing the rise in serial hCG in a normal pregnancy can be described as log linear (2, 16–18), quadratic (19, 20), or a combination of both, depending on gestational age (21, 22). Using a random-effects model and various statistical analysis methods, we precisely describe the hCG profiles of pregnant women who later developed GDM after conceiving through IVF and ultimately achieved live births. In this study, we restricted the sample to pregnancies with live births achieved through IVF technology. In addition, all maternal complications and other pregnancy complications, such as hypertension/gestational hypertension and thyroid disease/gestational thyroid diseases, were excluded. Previous studies have reported that pregnant women with higher hCG levels in the first trimester of pregnancy had a reduced risk for GDM (23), which is consistent with our results. In addition, the abnormal low or high level of hCG in the second trimester was also associated with other pregnancy complications such as gestational hypertension and preeclampsia (24–28) in previous studies focusing on gestational weeks 11–13^+6^ or later. Therefore, we excluded other complications that may affect hCG levels in the present study. To the best of our knowledge, no study has assessed the difference in hCG levels between women with and without GDM after IVF in early pregnancy.

A previous study (29) including 391 IVF cycles resulting in live birth reported that the initial hCG levels were lower in women with a BMI >30 kg/m^2^. Results of our study suggested that BMI had no effect on initial hCG levels, although this could be explained by the fact that there was only one patient with a BMI >30 kg/m^2^. It is also worth noting that the participants of the previous study were white. In contrast, they are Asians in the present study. Ethnicity has long been recognized as a major risk factor to the development of GDM (30), and the risk increases exponentially with increasing BMI (31). Obese, overweight patients are often asked to lose weight before starting an IVF cycle in order to improve the success rate. So, obesity is the most salient modifiable risk factor for the condition (32). According to the latest BMI classification standard (33), in the Asian population, BMI over 25 is defined as obesity. All participants in the present study, except one, had BMI less than 25 kg/m^2^.

Our data indicated that pregnancies achieved through IVF, and delivered a live birth, on average, demonstrated a relative hCG increase of 55% in 1 day and 211% in 2 days (slope, 0.173). The slowest rate of increase in our population of pregnancies was 19% in 1 day and 138% in 2 days. A retrospective cohort analysis reported a predicted increase of 1.50 (50% increase) in 1 day and 2.24 (124%) in 2 days of serum hCG in viable pregnancies achieved through use of IVF (8). In our study, the mean 1 day increase in hCG was similar to that reported in the literature (55% vs. 50%); however, the mean 2-day increase in hCG was faster (211% vs. 124%), which may be related to the exclusion of some other pregnancy complications that were associated with early hCG rise.

The initial hCG values on day 14 and day 16 post-oocyte retrieval in the non-GDM group were higher than those in the GDM group, which was consistent with a systematic review indicating that women diagnosed with GDM exhibit lower first trimester (11–13^+6^ weeks) hCG levels than those who remained normoglycemic (34). However, the exact mechanism remains unclear. A possible explanation is that, although blood glucose level in women with GDM is normal in early pregnancy, they may have some high-risk factors for gestational diabetes, such as a family history of diabetes or genetic susceptibility. These high-risk factors may cause a state of potential hyperinsulinemia. Hyperinsulinemia leads to reduced concentrations of insulin-like growth factor binding protein-1 (IGFBP-1) and glycodelin in the early stages of pregnancy, thereby interfering with the proliferation of trophoblasts (35). Furthermore, hyperinsulinemia may increase the level of plasminogen activator inhibitor-1 and induce villous thrombosis, thereby reducing blood supply and leading to trophoblastic hypoplasia (36). In twin pregnancies, the initial hCG values (days 14 and 16) in the non-GDM group were higher than those in the GDM group, although the difference was not statistically significant. It may result from the possibility that “twins” itself was a high-risk factor for GDM (10). The proportion of patients with low initial hCG levels was also higher in the GDM group, although the difference was not statistically significant. Furthermore, the OGTT may be very weakly and negatively correlated with initial hCG values. Therefore, a low initial hCG value may indicate a high risk for the development of GDM.

The present study devoted special attention to pregnant women with GDM and indicated that early hCG rise after IVF, whether GDM or non-GDM, could be characterized by quadratic and linear models. Our findings suggest that initial hCG values in non-GDM women were significantly higher than those in women who later developed GDM, with the exception of twin pregnancies. Low hCG values in early pregnancy may be a clue to help predict the likelihood of GDM in the subsequent gestation period.

## Data Availability Statement

The original contributions presented in the study are included in the article/supplementary material. Further inquiries can be directed to the corresponding authors.

## Ethics Statement

The studies involving human participants were reviewed and approved by the Ethical Committee of Women’s Hospital, Zhejiang University School of Medicine. Written informed consent for participation was not required for this study in accordance with the national legislation and the institutional requirements.

## Author Contributions

Y-HT and D-QC designed the project. L-FZ and Y-TL collected and analyzed the data. WW drafted the article. All authors contributed to the article and approved the submitted version.

## Funding

The work was sponsored by the National Key R&D Program of China (2018YFC1004302) and Major R&D projects of the Science and Technology Department of Zhejiang Province (2018C03010).

## Conflict of Interest

The authors declare that the research was conducted in the absence of any commercial or financial relationships that could be construed as a potential conflict of interest.

## Publisher’s Note

All claims expressed in this article are solely those of the authors and do not necessarily represent those of their affiliated organizations, or those of the publisher, the editors and the reviewers. Any product that may be evaluated in this article, or claim that may be made by its manufacturer, is not guaranteed or endorsed by the publisher.
